# Cs^+^ Promoting the Diffusion of K^+^ and Inhibiting the Generation of Newberyite in Struvite-K Cements: Experiments and Molecular Dynamics Simulation Calculations

**DOI:** 10.3390/ma17040814

**Published:** 2024-02-08

**Authors:** Difei Leng, Qiuyan Fu, Yunlu Ge, Chenhao He, Yang Lv, Xiangguo Li

**Affiliations:** State Key Laboratory of Silicate Materials for Architectures, Wuhan University of Technology, Wuhan 430070, China; lengdifei@126.com (D.L.); fuqiuyan01@126.com (Q.F.); yunluge01@126.com (Y.G.); hechenhao01@126.com (C.H.)

**Keywords:** struvite-K cements, newberyite, Cs immobilization

## Abstract

Struvite-K cements, also called magnesium potassium phosphate cements (MKPCs), are applicable for particular applications, especially the immobilization of radioactive Cs^+^ in the nuclear industry. This work focuses on how Cs^+^ affects the hydration mechanism of struvite-K cements because newberyite and brucite in the hydration products are deemed to be risky products that result in cracking. Experiments and molecular dynamics simulations showed that Cs^+^ promoted the diffusion of K^+^ to the surface of MgO, which greatly facilitates the formation of more K-struvite crystals, inhibiting the formation of newberyite and brucite. A total of 0.02 M Cs^+^ resulted in a 40.44%, 13.93%, 60.81%, and 32.18% reduction in the amount of newberyite and brucite, and the Cs immobilization rates were 99.07%, 99.84%, 99.87%, and 99.83% when the ratios of Mg/P were 1, 3, 5, and 7, respectively. This provides new evidence of stability for struvite-K cements on radioactive Cs^+^ immobilization. Surprisingly, another new crystal, [CsPO_3_·H_2_O]_4_, was found to be a dominating Cs-containing phase in Cs-immobilizing struvite-K cements, in addition to Cs-struvite.

## 1. Introduction

During the operation of nuclear power plants, nuclear waste disposal will be generated. Normally, these nuclear wastes would be treated by dividing them into four procedures. (1) These nuclear wastes would be dissolved in boiling nitric acid to extract valuable uranium and plutonium (PUREX process), leaving a large amount of high, medium, and low levels of liquid radioactive waste (LRW). (2) These high-temperature LRWs would be stored in a pool for a cooling process. (3) These cooled LRWs would be moved to specialized drying facilities for drying in order to reduce the volume of LRWs. (4) Concentrated LRWs would be blended with borosilicate glass at high temperatures and finally form a glass phase [1,2,3,4,5,6,7,8,9]. The safe treatment of these LRWs is one of the leading problems in the nuclear industry [10,11,12,13,14,15,16,17]. Cs^+^ is one of the main radioactive ions in low-level and medium-level LRWs [18,19,20]. Traditionally, they are mixed with borosilicate glass under high-temperature conditions to produce a solidified body in the form of glass [11]. The advantage of this method is that the volume of the solid is significantly reduced, but the disadvantage is the volatilization of Cs under 1500 °C when people find these high-activity fission products, such as radioactive ^134^Cs and ^137^Cs, end up in the off-gas stream that is collected during vitrification [21]. The method of using synthetic zeolite, such as Cs, as an adsorbent is also worthy of attention, and it is one of the most promising Cs adsorbents [22]. Its disadvantage is that high-temperature sintering is required, and the volatility of CsCl at high temperatures needs to be considered; thus, sintering needs to be carried out at the lowest temperature and in the shortest time, if possible. The latest spark plasma sintering (SPS) technology has effectively solved this shortcoming [23]. Ordinary Portland cement is also a traditional immobilization matrix, which has the advantages of a simple process and low cost, but its disadvantages are a high ionic leaching rate, a long curing time, and a large volume [24,25]. The use of struvite-K cements to immobilize Cs^+^ is generally regarded as a promising method and has many advantages, such as the inhibition of ionic volatilization under high temperature, rapid setting, high early strength, and a low ionic leaching rate, so this method has been widely studied [21,26,27,28,29]. Struvite-K cements, also known as chemically bonded phosphate ceramics (CBPCs), are a new type of cement [30,31,32,33,34] that are generated by an ionic reaction of dead burned MgO and KH_2_PO_4_ with water:

MgO  +  KH_2_PO_4_  +  5H_2_O → MgKPO_4_·6H_2_O (1)

Struvite-K cement is a special cement with rapid curing and great developing strength, and it is usually used in emergency bridge or road repair works [28]. It is also used for the immobilization of radioactive nuclides [10,35]. Other related issues are the recovery of potassium and phosphorus from urines in the form of K-struvite [36,37,38,39,40,41], or the recovery of nitrogen and phosphorus from municipal sewage/sludge in the form of struvite [40,42,43,44,45,46,47]. The effect of additional cations on the struvite-K cement system is believed to be critical. Gaoet. al deemed that the presence of Na^+^, NH^4+^, and Ca^2+^ evidently inhibited the crystallization of K-struvite [36]. Liu et al. believed that the Ca/Mg ratio not only affected recovery efficiency of phosphorus and solution pH but also affected crystal characterization, including size, purity, and shape [48]. Struvite could promote the precipitation of heavy metals (Cu, Ni, Pb, Zn, Mn, Cr(III)), and heterogeneous nucleation of heavy metal hydroxides on struvite was detected [49]. Zhou et.al were convinced that struvite-K cements could deal with arsenic remediation in lollingite-polluted environments [50]. Pang et al. proposed that K-struvite whisker could enhance Pb immobilization in MKPC [51].

Examining Cs^+^ in the struvite-K cement system, Arun S. Wagh et al. deemed that the struvite structure of struvite-K was an excellent host of radioactive cesium, and the volatility problem of Cs^+^ could be avoided by this method [21]. Laura J. Gardner et al. believed blended struvite-K cement binders could have an ideal encapsulation up to at least 1200 °C [29]. The struvite-K cement method showed effective solidification performance, higher mechanical strength, and greater waste encapsulation capacity [26,27,52]. There are few in-depth studies on the immobilization mechanism of Cs^+^ in the KMPC system; most studies point out that Cs^+^ could be mineralized in K-struvite crystals, and Cs could replace part of the K^+^ sites (Cs-struvite is the mineral name of MgCsPO_4_·6H_2_O):

MgKPO_4_·6H_2_O  +  xCs^+^ → MgCs_x_K_(1−x)_PO_4_·6H_2_O + xK^+^ (2)

or:

MgKPO_4_·6H_2_O + Cs^+^ → MgCsPO_4_·6H_2_O + K^+^ (3)

Regarding this immobilization mechanism, Leng et al. used the DFT method to calculate the mechanism of Cs^+^ immobilization by K-struvite in an aqueous solution and proposed the Aqueous Solution Substitution Forming Energy (ΔE_aq_) as the criterion to confirm it [35].

The vast majority of studies are from the perspective of struvite-K cements immobilizing Cs^+^. Few studies stand on another perspective; that is, Cs^+^ affects the hydration mechanism and hydration products of struvite-K cements as an additive. Tao et al. performed basic research on this aspect and emphasized that Cs^+^ participated in the hydration reaction of CBPC, which reduced the crystallinity of K-struvite. The compressive strength of the solidified struvite-K cement samples with Cs^+^ could meet the requirement [52]. In addition, newberyite, one of the hydration products of struvite-K cements, is considered to be an important risk factor for the expansion and cracking of struvite-K cements, and may pose a potential risk to the solidified matrix [53]. However, the effect of introducing Cs^+^ on newberyite has not been studied.

In addition to Cs-struvite, regarding the form of Cs^+^ in the struvite-K cement system, Cs_3_PO_4_ is considered another Cs-containing crystal [21,33,54] because some hydration products are made of Cs, P, and O but not Mg by energy-dispersive X-ray spectroscopy. In this work, [CsPO_3_·H_2_O]_4_ (Tetracaesium cyclo-tetrametaphosphate tetrahydrate) accounts for these hydration products rather than Cs_3_PO_4_.

In summary, it is important to understand how Cs^+^ affects the hydration mechanism of struvite-K cements, as Cs^+^ might result in changes in the types and quantities of hydration product crystals, ultimately affecting people’s understanding and evaluation of struvite-K cement as a kind of Cs-immobilized material. This is the starting point of this work, especially regarding whether the introduction of Cs^+^ would increase the inherent potential cracking risk of the struvite-K cement system. This is particularly important for the immobilization of radioactive materials, as even minor cracks could greatly increase the exposure area of radioactive nuclides, thereby increasing the risk of their diffusion into the environment. The effect of Cs^+^ on the hydration mechanism of struvite-K cements, especially the change in the population of hydration products, was investigated in this work. This study emphasizes the fact that Cs^+^ would inhibit the generation of newberyite and brucite in struvite-K cements. Molecular dynamics (MD) simulation calculations were used to gain insight into the effects of Cs^+^ on the hydration process of struvite-K cements, especially the diffusion of K^+^. Furthermore, it is found, for the first time, that Cs^+^ also exists in the form of [CsPO_3_·H_2_O]_4_, in addition to Cs-struvite, in the struvite-K cement system.

## 2. Experimental Methodology

### 2.1. Experimental Method

#### 2.1.1. Raw Materials Procedures

**Figure 1:**
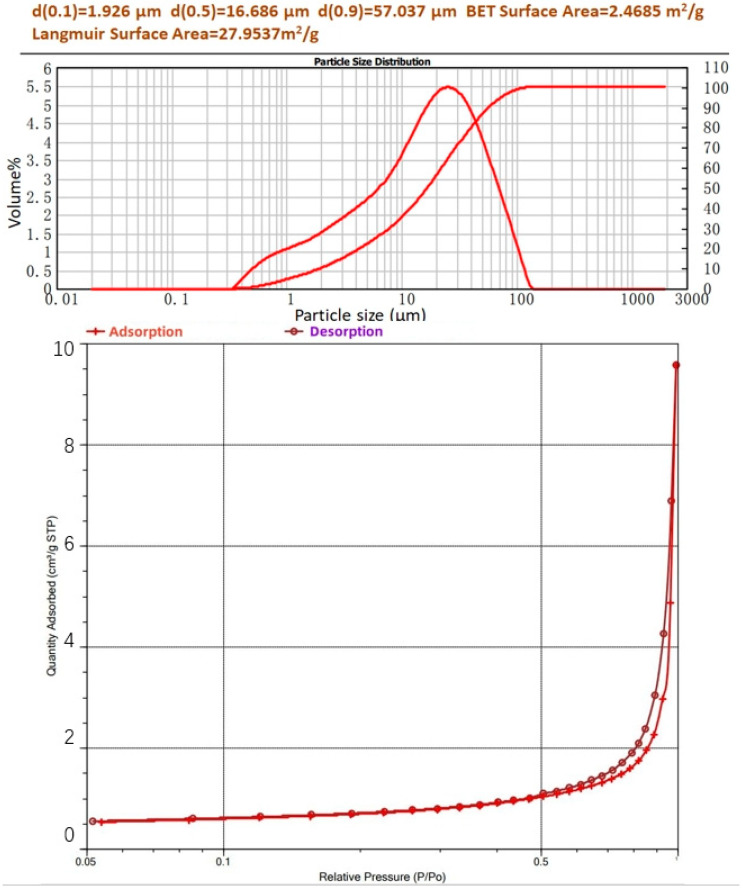
PSD, the integral of PSD, BET surface area, and Langmuir surface area of the dead burned MgO.

In this experiment, industrial product dead burned magnesium oxide (MgO, from Qiaoxu Magnesium Material Factory, Yingkou, China), analytical reagent potassium dihydrogen phosphate (KH_2_PO_4_, from Shanghai Aladdin, Shanghai, China), analytical reagent CsCl (from Shanghai Aladdin, Shanghai, China), and ultrapure water were used to prepare struvite-K cement hydration products. Existing studies have shown that the immobilization of Cs has nothing to do with whether the isotope is radioactive, so it is appropriate to use a non-radioactive Cs source [21]. The chemical composition of dead burned magnesium oxide was determined by X-ray fluorescence (XRF) oxide analysis (Table 1), and the particle size distribution (PSD) and specific surface area were analyzed by a laser particle analyzer (Mastersizer 2000 from Malvern Panalytical, Malvern, UK) and fully automatic specific surface area and porosity analysis (ASAP 2460 from Micromeritics, Norcross, GA, USA) in Figure 1. The citric acid reactivity time of the dead burned MgO was 7200 s.

#### 2.1.2. Specimen Preparation

In this experiment, KH_2_PO_4_ and CsCl were pre-dissolved in ultrapure water and subsequently reacted with dead burned MgO to produce struvite-K cement hydration products. The water-to-total-solids ratio of the experiment was 10 (in terms of the ratio of water and MgO, w/s = 10). The molar ratios of dead burned MgO and KH_2_PO_4_ (Mg/P) were 1:1, 3:1, 5:1, and 7:1, and were denoted as MP1, MP3, MP5, and MP7. The molar ratios of the dead burned MgO and KH_2_PO_4_ were 1:1, 3:1, 5:1, and 7:1, with a concentration of 0.02 M Cs^+^, denoted as Cs1, Cs3, Cs5, and Cs7. The specific ratios are shown in Table 2. There was no retarder in this work. These samples were sealed with plastic films after stirring for 30 min with a magnetic stirrer (pH values started to be tested during this time) and cured at 298.15 K for 21 days. Solid and leachate were separated by a negative pressure suction filter device.

#### 2.1.3. Analysis Method

The pH of the solution was monitored by a pH meter (pHS-3C). Concentrations of K^+^ and Cs^+^ in leachate were analyzed by an atomic absorption spectrometry instrument (AAS, CONTRAA-700 from Analytik Jena AG, Jena, Germany), and concentrations of P were analyzed by Inductively Coupled Plasma–Optical Emission Spectrometry (ICP-OES, Prodigy 7 from Hudson, NH, USA). Rietveld X-ray diffraction refinement was adopted to quantitatively study the crystal phases of the samples (XRD, Empyrean from Malvern Panalytical, Almelo, The Netherlands, 10° < 2θ < 65°, 1.83°/min). The elemental distribution and morphology of the samples were analyzed by energy-dispersive X-ray spectroscopy (EDS, ESCALAB 250Xi from Thermo Fisher Scientific Co., Waltham, MA, USA) and a scanning electron microscope (SEM, JSM-IT300 from JEOL Ltd., Akishima, Japan).

### 2.2. Computational Method

In this work, Gromacs was used to run all molecular dynamics (MD) simulation calculations [55,56,57,58,59,60]. Every MD simulation calculation runs an NPT process for 80 ps, and then an NVT process for 20 ns. A velocity-rescale thermostat was used every 0.2 ps. Gromos54a7.ff was the main force field, and kbff20.ff was adopted to describe the non-bond parameters of Cs^+^, K^+^, and Mg^2+^. Sobtop was used to obtain the non-bond parameters of MgO, H_2_PO_4_^−^, and HPO_4_^2−^. The Ewald sum method was adopted to deal with long-range electrostatics under 1.2 nm. The timestep was set to 1 fs, and the simulated temperature was set to 320 K.

**Figure 2:**
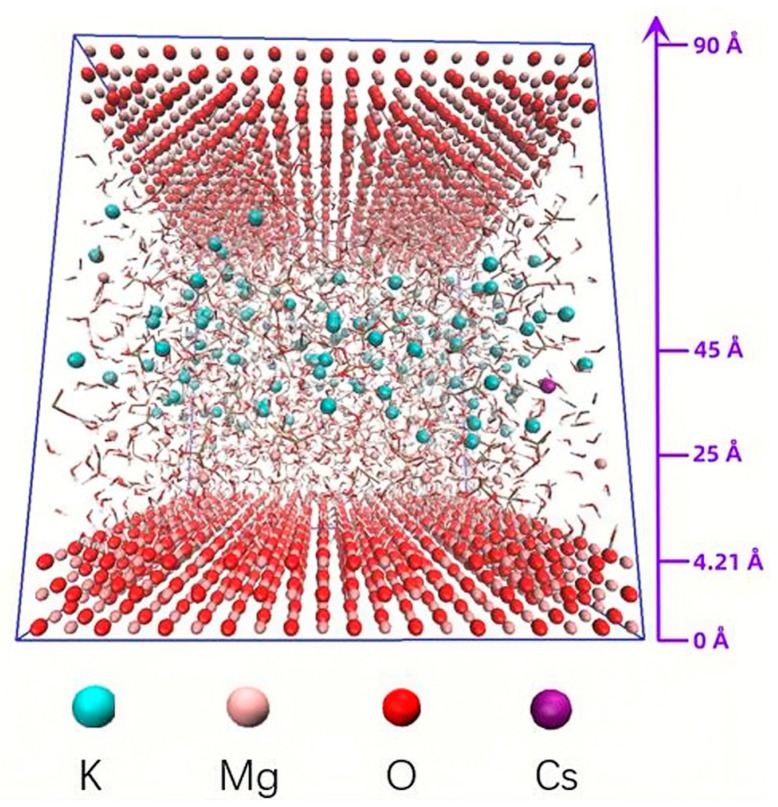
Sample graph for Cs1 of the initial distribution of ions in MD models.

The focus of molecular dynamics simulation was to investigate the effect of Cs^+^ on the ability of K^+^ to diffuse to the surface of MgO. In order to avoid contingency, Packmol [61] was used to construct a model with a Mg/P of 1, 3, and 5, respectively, for MD calculation (see Figure 2 and Table 3). The initial K^+^ and Cs^+^ were located in the aqueous solution near the middle interface of the z-axis (25–45 Å), far away from the surface of magnesium oxide (0–4.21 Å). The dissolved Mg^2+^ (4.21–25 Å) was closer to the magnesium oxide. The amounts of water molecules, HPO_4_^2−^ and H2PO_4_^−^, in the models were determined by the measured pH and the Henderson–Hasselbalch equation:

pH-pK_a_ = lg ([H_2_PO_4_^−^]/[HPO_4_^2−^]) (4)

## 3. Results and Discussion

### 3.1. pH Value and Concentrations of Potassium and Phosphorus in Leachate

**Figure 3:**
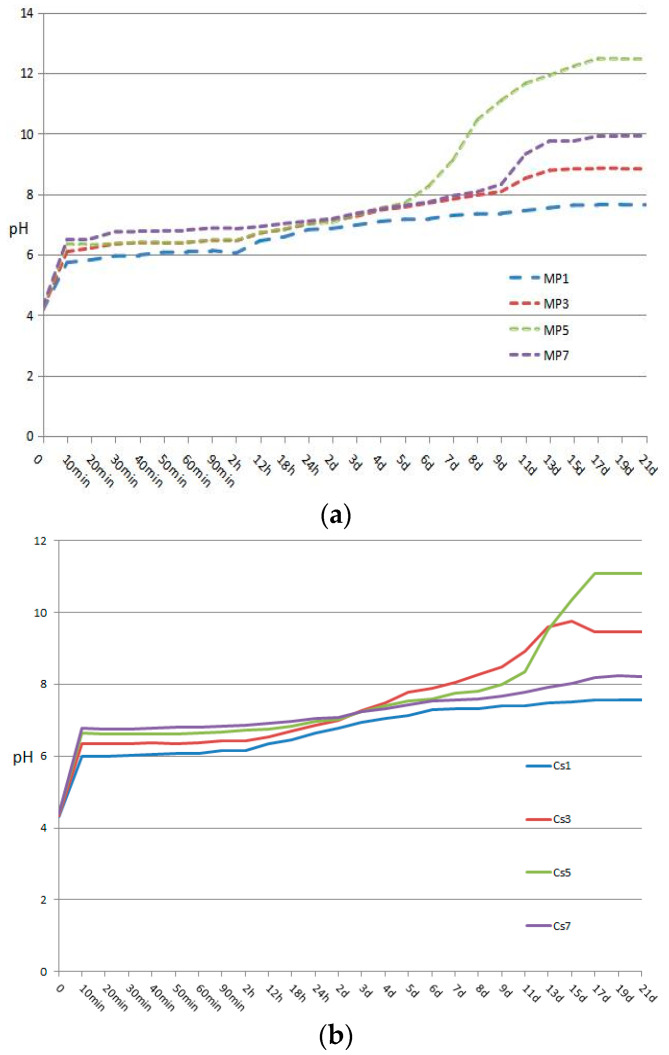
pH of the solution of the struvite-K cement systems. (**a**) pH of the solutions of MP1, MP3, MP5, and MP7; (**b**) pH of the solutions of Cs1, Cs3, Cs5, and Cs7.

The importance of pH for acid–base reactions in solutions is obvious. When the pH is between 4 and 6, the hydration product is newberyite, and K-struvite begins to appear when pH > 6 [53]. Because the CsCl solution was alkaline, the initial pHs of all Cs-containing solutions were higher than the corresponding blank groups. However, pH values of all Cs-containing solutions were already lower than the corresponding blank groups at no more than 24 h (Figure 3). Since Cs-struvite was considered to be thermodynamically easier to generate than K-struvite [35], the requirement of pH for Cs-struvite generation was lower. After Cs-struvite was formed, it might act as a seed to promote the formation of K-struvite. The Cs^+^ in the solution provided new favorable conditions for the formation of K-struvite, thereby reducing the pH value originally required for the formation of K-struvite.

**Figure 4:**
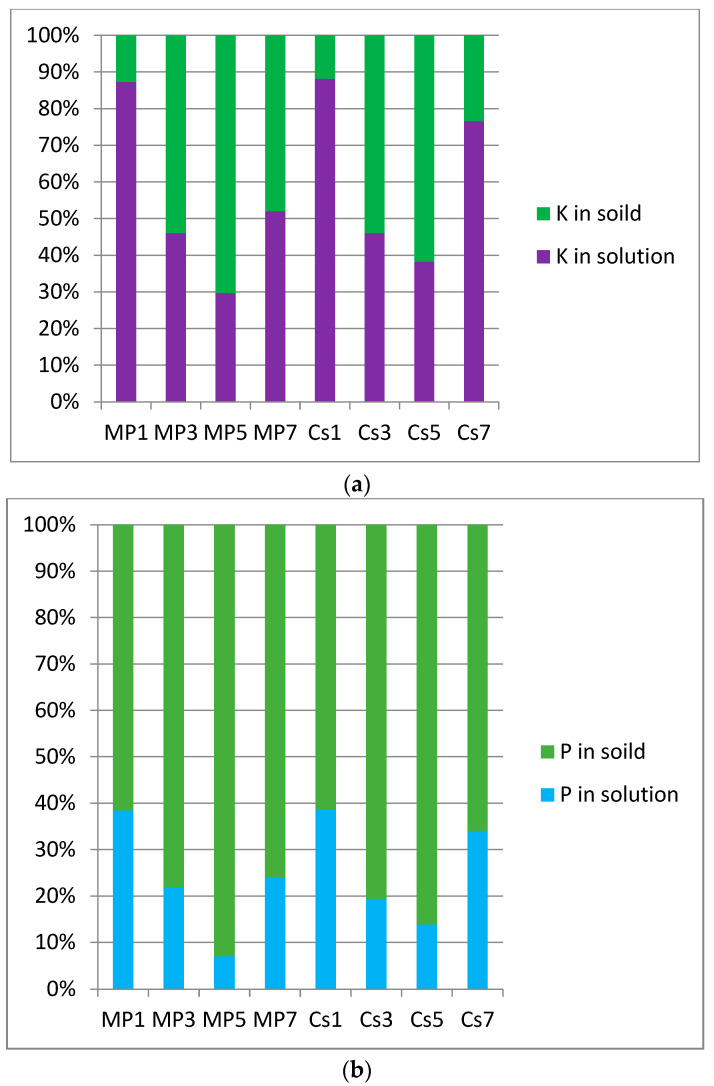
Population of K^+^ and P in the solution and solids of the samples. (**a**) Population of K^+^ in the solution and solids of the samples. (**b**) Population of P in the solution and solids of the samples.

To study the effect of Cs^+^ on the hydration process of struvite-K cements, AAS was used to measure the concentrations of K^+^ and Cs^+^ in each group of liquid samples, and ICP-OES was used to measure the concentration of P in liquid samples. Concentrations of K^+^ in leachate were analyzed by an atomic absorption spectrometry instrument (CONTRAA-700), and concentrations of P were analyzed by Inductively Coupled Plasma–Optical Emission Spectrometry (Prodigy 7). It is easy to observe in Figure 4 that Mg/P had a key control effect on the residual amount of K and P in the solution, and their changing trends were consistent, namely:

MP1(K) > MP7(K) > MP3(K) > MP5(K), MP1(P) > MP7(P) > MP3(P) > MP5(P);

Cs1(K) > Cs7(K) > Cs3(K) > Cs5(K),Cs1(P) > Cs7(P) > Cs3(P) > Cs5(P).

Assume that Cs-struvite, which was thermodynamically easier to generate, would be generated before K-struvite at pH < 6, acts as a seed to promote K-struvite. If struvite (both K-struvite and Cs-struvite) was generated, Cs^+^ should be helpful to reduce the tendency of phosphorus to remain in the solution. However, in fact, the residual rate of phosphorus in the Cs group was basically unchanged from the value and trend of the blank group. In this study, it is believed that the induction effect of Cs^+^ was similar to the buffering effect of an acid–base buffer pair. On the one hand, the presence of Cs^+^ enabled the formation of Cs-struvite at pH < 6 and induced the formation of K-struvite as a seed crystal. This facilitated the conversion of phosphates from the solution to solids. On the other hand, the induction of Cs^+^ made struvite-K cements generate more Cs-struvite and K-struvite cementitious phases in the early stage of hydration. These cementitious phases covered the surface of unreacted MgO particles and wrapped them more densely, including a larger amount of unreacted MgO. Corresponding phosphorus-containing acid radical in the solution could not enter the solid, thereby increasing the tendency of phosphorus to remain in the solution. The effect of Cs^+^ on the tendency of K^+^(aq) to remain was similar to the effect on P, which was also dominated by buffering. Apparently, Cs^+^ basically had no effect on the tendency of K^+^ and phosphorus in the solution to leave the solution and enter the solid. But, in fact, Cs^+^ changed the hydration process of struvite-K cements, which would inevitably affect the type and population of hydration products.

Immobilization rates of Cs are given in Table 4. It can be seen that each Mg/P ratio struvite-K cement system had a good immobilization effect on Cs^+^ with each Mg/P ratio. Cs^+^ had a strong tendency to move out of the solution and into solid in the struvite-K cement system.

### 3.2. Quantitative Analysis of Hydration Products of Struvite-K Cements

**Figure 5:**
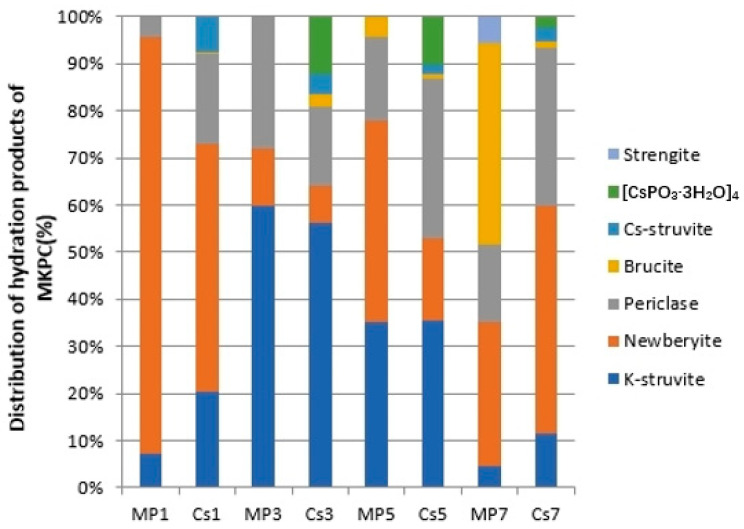
Crystal phase types and the population of hydration products in the struvite-K cement system.

**Figure 6:**
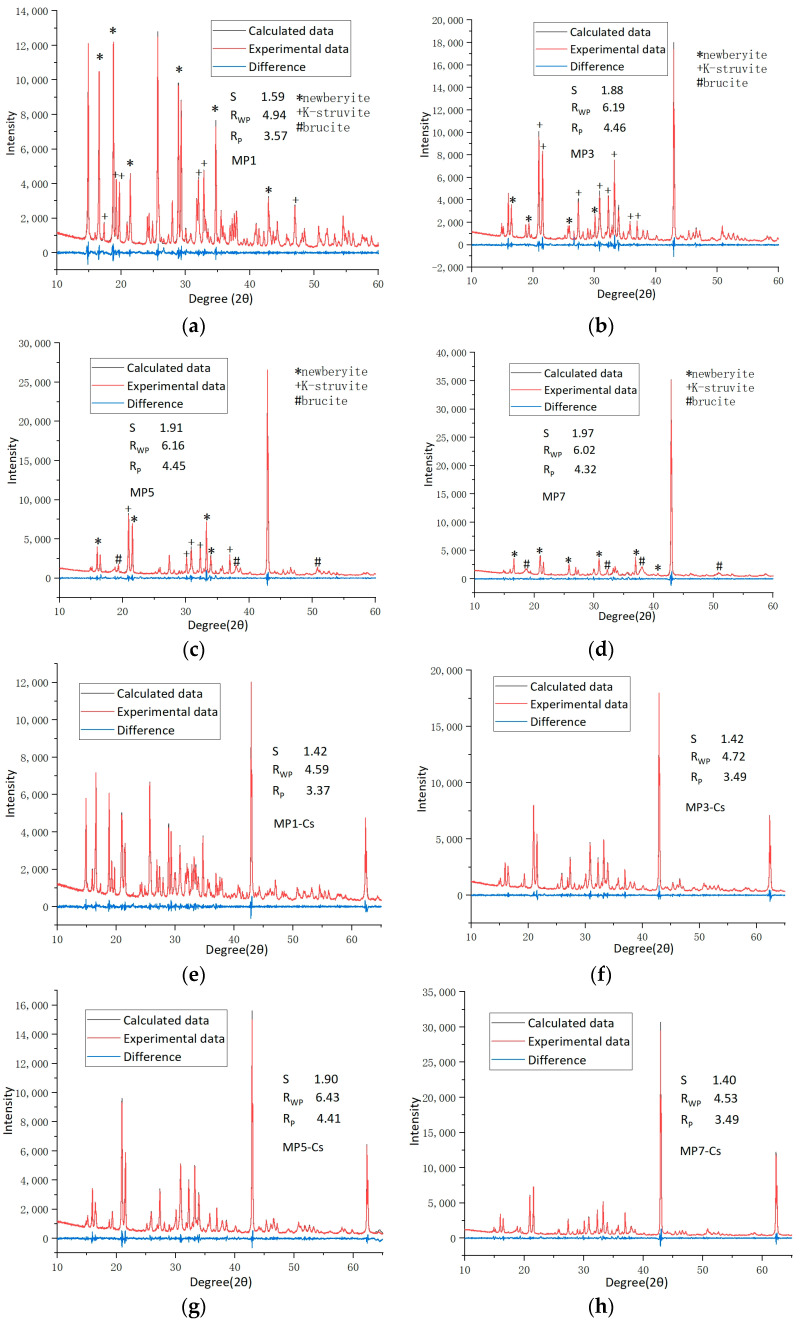
X-ray diffraction patterns for (**a**) MP1, (**b**) MP3, (**c**) MP5, (**d**) MP7, (**e**) Cs1, (**f**) Cs3, (**g**) Cs5, (**h**) Cs7. The molar ratios of dead burned MgO and KH_2_PO_4_ (Mg/P) were 1:1, 3:1, 5:1, 7:1, denoted as MP1, MP3, MP5, MP7. The molar ratios of dead burned MgO and KH_2_PO_4_ were 1:1, 3:1, 5:1, 7:1 with a concentration of 0.02M Cs^+^, denoted as Cs1, Cs3, Cs5, Cs7.

Rietveld XRD refinement was adopted to quantitatively study the effect of Cs^+^ on the type and distribution of hydration products of struvite-K cements under different Mg/P, and the population of crystal phases was shown in Figure 5. Detailed XRD data are shown in Figure 6.

The first focus is on the ratio of [K-struvite + Cs-struvite] in the hydration products of struvite-K cements. For the hydration process of struvite-K cements, according to Equation (1), Mg/P = 1:1 is stoichiometric and produces K-struvite. Unfortunately, since the surface of the MgO particles gradually dissolves and participates in the formation of K-struvite, the newly generated K-struvite would also densely cover the MgO particles and be completely wrapped; that is to say, there would always be MgO that could not participate in the reaction and would thus be forced to become the de facto aggregate [28]. Typically, a Mg/P molar ratio over 6:1 would lead to a harmful effect on compressive strength [62,63,64]. Therefore, MP7 and Cs7 are for simple reference only in this work. In conclusion, for the struvite-K cement system, since K-struvite is the predominant cementitious phase, generating as much K-struvite as possible would be considered beneficial for compressive strength. When Cs^+^ is introduced in the struvite-K cement system, we also think that Cs-struvite is the cementitious phase due to its similar structure to K-struvite. By comparing [MP1, Cs1], [MP3 and Cs3], and [MP5, Cs5], it could be clearly seen that the addition of Cs^+^ is beneficial to the formation of cementitious phase [K-struvite + Cs-struvite], which strengthens the cementing properties of the struvite-K cement system. At the same time, with a change in the cementation phase ratio, the content of another main crystalline phase is also easily noticed, which is the next focus, newberyite.

The second focus is the amount of newberyite being generated. Long-neglected newberyite is one of the hydration products of struvite-K cements. It was not until around 5 years ago when the formation mechanism of K-struvite was studied that newberyite was confirmed to be an important intermediate in the formation of K-struvite. In addition, newberyite in struvite-K cement hydration products was considered to be a factor that could cause the potential risk of expansion and cracking of struvite-K cements [53]. For [MP1, Cs1], [MP3, Cs3], and [MP5, Cs5], the presence of Cs^+^ in an aqueous solution apparently inhibited the formation of newberyite. This suggested that regarding Cs^+^ as an admixture, Cs reduced the risk of expansion cracking of struvite-K cements, which was beneficial for the immobilization of radioactive ions by struvite-K cements. In addition, Cs^+^ could also inhibit the formation of brucite under Mg/P = 1:7. Brucite was also a risk factor for expansion and cracking in the struvite-K cement system [53]. A total of 0.02 M Cs^+^ resulted in a 40.44%, 13.93%, 60.81%, and 32.18% reduction in the amount of newberyite and brucite when the ratios of Mg/P were 1, 3, 5, and 7, respectively.

The third focus is the existence of a Cs-containing crystalline phase, [CsPO_3_·H_2_O]_4_, which has never been discovered in the hydration products of struvite-K cement. There is only one reference available about this crystalline phase, which addresses its first discovery in 1986 [65]. CsPO_3_·H_2_O is prepared by slowly adding P_4_O_10_ stoichiometrically to an appropriate amount of a Cs_2_CO_3_ aqueous solution at 273 K and slowly evaporating at room temperature. Similar tetrametaphosphate crystals, such as [NaPO_3_·H_2_O]_4_ and [KPO_3_·H_2_O]_4_, could also be prepared by the corresponding carbonate solution [66,67]. After adding P_4_O_10_, they are prepared by slow evaporation or by adding a large excess ethyl alcohol. They are usually gels, and adding an appropriate amount of corresponding hydroxide to the solution would increase the crystallinity of the gel. In other words, [APO_3_·H_2_O]_4_ (A = Cs, K, Na) seems to be soluble, according to the references [68]. The presence of CsPO_3_·H_2_O in the struvite-K cement system came as a surprise, as it implied the presence of an insoluble simple ionic mineral of alkali metal, which was very rare. This meant that there were few Cs^+^ in the solution but lots of K^+^. Naturally, we tried to explain this phenomenon by K_sp_ theory but found that it was hard. Why did Cs^+^ prefer a solid rather than an aqueous solution? This is another interesting question. All in all, combined with the extremely low Cs concentration in the solution by the AAS test, Cs^+^ seemed to have a stronger tendency to enter a solid matrix than an aqueous solution. In addition to Cs-struvite, Cs^+^ prefers to become [CsPO_3_·H_2_O]_4_ rather than Cs^+^_(aq)_. The struvite-K cement system is an ideal matrix for Cs^+^ immobilization.

### 3.3. SEM and EDS Analysis

**Figure 7:**
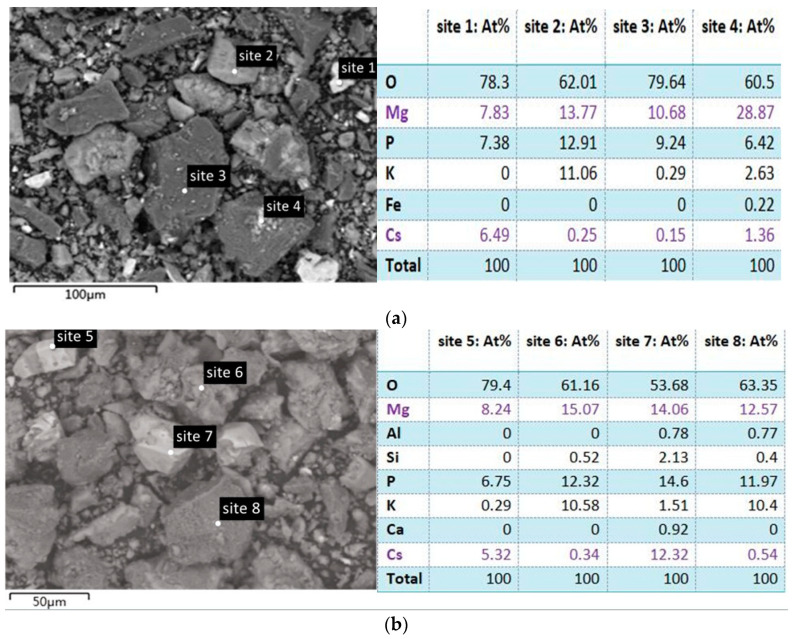

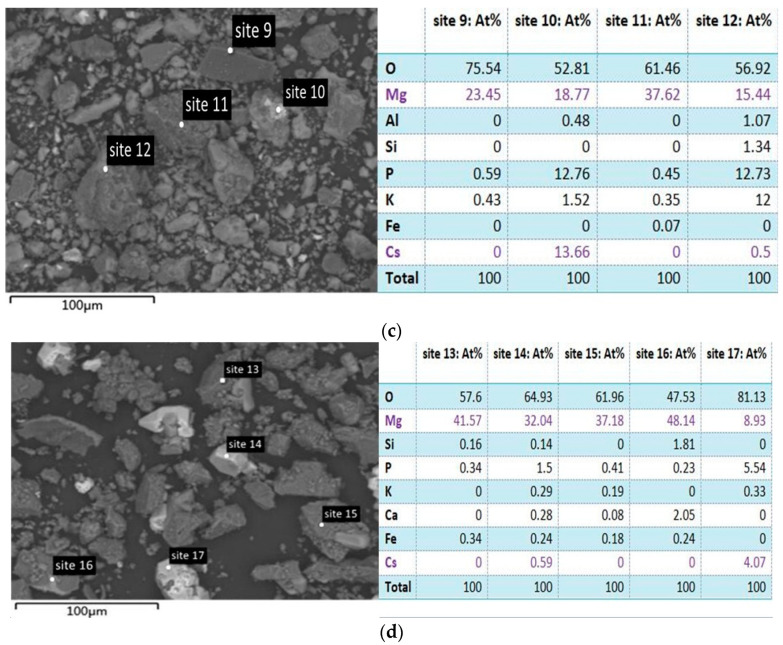
SEM and EDS patterns of struvite-K cement hydration products for (**a**) Cs1, (**b**) Cs3, (**c**) Cs5, (**d**) Cs7. At% of Mg and Cs are in purple in order to evaluate the types of Mg-containing minerals and the occurrence status of Cs.

To understand the effect of Cs^+^ on struvite-K cement hydration products, SEM and EDS were used to observe the morphology and element distribution of relevant samples. The elemental distribution and morphology of the products were analyzed by energy-dispersive X-ray spectroscopy (ESCALAB 250Xi) and a scanning electron microscope (JSM-IT300). As seen in Figure 7, the Cs-containing areas were all biased toward the bright field phase. Thus, Cs in the struvite-K cement system existed in some solids with steep surface morphology, and there were no exceptions in all SEM and EDS test results, including sites 1, 4, 5, 7, 10, 14, and 17. Prismatic solids of moderate brightness were mainly K-struvite, such as sites 2, 6, 8, and 12. Newberyite existed in the form of large dark-field phase bulk, such as sites 3 and 11. K or Cs are not simultaneously lacking in (b), indicating Cs^+^ had the most obvious inhibitory effect on the formation of newberyite under Mg/P = 3:1.

Assuming that Cs existed only in the form of Cs-struvite (MgCsPO_4_·6H_2_O) in the struvite-K cement hydration products, the content of Cs should not be higher than Mg. However, for each group of samples, if the Cs content of a certain site was relatively high, then the Mg content of the site would be relatively low, which indicated the existence of [CsPO_3_·H_2_O]_4_ in the hydration product.

### 3.4. The Effect of Cs^+^ on the Hydration Mechanism and Hydration Products of Struvite-K Cements

Generally, the chemical kinetics of the hydration reaction of struvite-K cements is mainly controlled by the dissolution of the dead burned MgO, which could increase the pH of the system. In addition to the Mg/P ratio, the activity of the dead burned MgO in the raw material is also a decisive factor, including the degree of sintering and the specific surface area. The reaction time of citric acid could be used to express the activity of the raw dead burned MgO.

With the dissolution of MgO, the pH of the solution rose slowly and went through three stages in a conventional struvite-K cement system [53]. Newberyite started to be generated in the solution when 4 < pH < 6. Then, newberyite was largely generated in the solution, and a small amount of K-struvite was generated when 6 < pH < 7. K-struvite was largely formed in the solution when pH > 7.

Cs^+^ changed the hydration kinetics of struvite-K cements, as Cs-struvite could be formed at lower pH than K-struvite and then act as a seed to promote the formation of K-struvite. Namely, MgO was transferred to cementitious phases. On the other hand, the excessively fast generation rate of Cs-struvite and K-struvite made it denser to encapsulate the unreacted MgO, which inhibited the further transformation of MgO to the cementitious phase. All in all, Cs^+^ had a buffering effect similar to a buffer solution in the struvite-K cement system.

Due to the change in reaction kinetics, Cs^+^ also affected the distribution of hydration products of struvite-K cements, inhibiting the formation of newberyite and brucite significantly. Newberyite and brucite were considered as phases with potential cracking risk. Cs^+^ made the formation of Cs-struvite and K-struvite more rapid, covering the surface of MgO that had not participated in the reaction more quickly, preventing the further dissolution of MgO and converting MgO into newberyite and brucite. In addition, Cs^+^ seemed to have a very strong tendency to leave the solution and enter the solid in the struvite-K cement system. Even if Cs-struvite could not be formed, [CsPO_3_·H_2_O]_4_ would be formed.

### 3.5. Effect of Cs^+^ on the Diffusion Kinetics of K^+^

**Figure 8:**
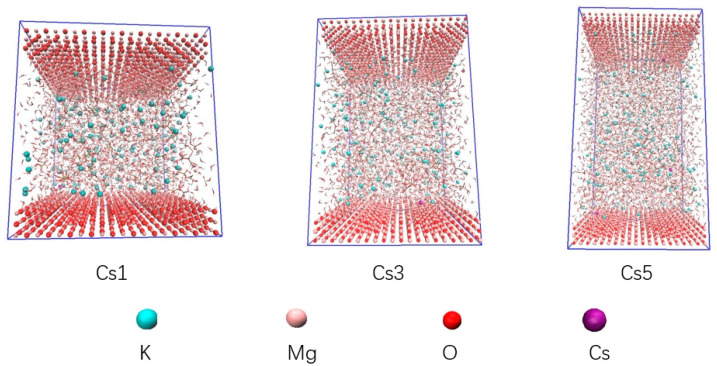
Flash of MD simulation process.

**Figure 9:**
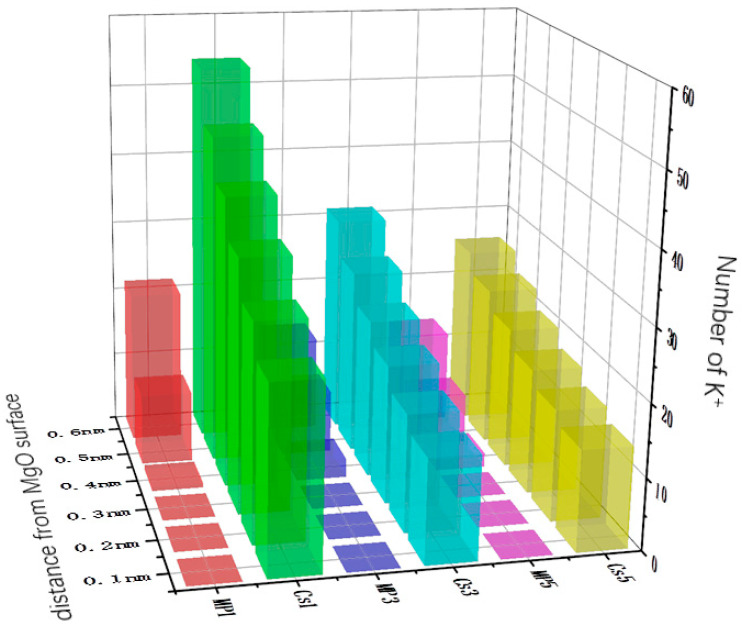
Number of K^+^ in different distances from the MgO surface.

Previous DFT calculations studies have shown that Cs-struvite is easier to generate than K-struvite and are the parts involving the chemical reaction that generates the struvite phase. However, prior to this process, before the chemical reaction occurs, K^+^ would diffuse in an aqueous solution near the MgO surface. At the same time, there is also dissolved Mg^2+^ near the MgO surface. K^+^, Mg^2+^, and PO_4_^3−^ react on the MgO surface to form K-struvite (MgKPO^4^·6H^2^O). The reason to emphasize the diffusion of K^+^ is that phosphorus-containing Mg^2+^ could form newberyite without K^+^ in water, namely MgHPO_4_·3H_2_O. If the extent of diffusion of K^+^ to the surface of the MgO is too low before the chemical reaction takes place, a large amount of newberyite without K^+^ will be formed, which will eventually cause the cracking of the entire cement system. MKPC is a fast-setting cement, which could be completely reacted in a few minutes, so it is difficult to observe the ion diffusion stage before the chemical reaction of the system by conventional means. Fortunately, molecular dynamics (MD) simulations do a good job of simulating the diffusion phase before a chemical reaction occurs. Figure 8 shows the flash of the MD simulation. The MD simulation animation shows that with a Mg/P of 1, 3, and 5, a low concentration of Cs^+^ could greatly promote the diffusion of K^+^ in the solution to the MgO surface. In order to quantify the effect of Cs^+^ on K^+^ diffusion kinetics, the homogenized number density of the MD simulation system was counted, and the number of K^+^ up to 0.1 nm, 0.2 nm, 0.3 nm, 0.4 nm, 0.5 nm, and 0.6 nm above the surface of MgO was obtained by its integral. As shown in Figure 9, a small amount of Cs^+^ could greatly increase the degree of K^+^ diffusion to the surface of MgO. The presence of Cs^+^ increased the amount of K^+^ by 151.4%, 131.3%, and 95.4% at a Mg/P of 1, 3, and 5, respectively, for up to 0.6 nm on the MgO surface. This provides conditions for the generation of K-struvite, inhibiting the generation of newberyite and reducing the risk of cracking in the MKPC system. This is beneficial for preventing the MKPC system from being exposed to the environment and continuously releasing Cs^+^ after cracking.

## 4. Conclusions

In this work, the effect of Cs^+^ on the products and mechanism of hydration of struvite-K cements was investigated. The main conclusions are as follows:

By influencing the hydration mechanism of struvite-K cements, Cs^+^ inhibits the formation of newberyite and brucite in the product, thereby reducing the potential cracking risk of the struvite-K cement immobilization matrix.

Cs^+^ affects the hydration process of struvite-K cements in three ways. Firstly, Cs^+^ greatly promotes the diffusion of K^+^ to the surface of MgO before the chemical reaction occurs. This inhibits the formation of newberyite. Secondly, Cs^+^ promotes the formation of K-struvite and Cs-struvite in the early hydration phase and promotes the transformation of MgO into the cementitious phase. Thirdly, the rapid formation rate of K-struvite and Cs-struvite makes it denser to wrap the unreacted MgO, which inhibits further transformation of MgO into the cementitious phase. The combined effect of these two inhibits the formation of newberyite and brucite.

For a wider scientific community of radiochemistry, it is an important issue to evaluate whether solid materials used for immobilized nuclides are at risk of cracking during the long term, as even minor cracks could greatly increase the exposure area of radioactive nuclides, thereby increasing the risk of their diffusion into the environment. This work provides a way to evaluate and improve potential cracking risks by considering the impact of nuclides on the properties of the solidified matrix itself. Obviously, using other additives to inhibit its potential cracking risk is also a feasible approach. More research needs to be conducted.

In addition, [CsPO_3_·H_2_O]_4_ is also the main crystalline phase in the struvite-K cement system, in addition to Cs-struvite.

## Figures and Tables

**Table 1:** XRF oxide analysis of the dead burned MgO.

Compound	wt%
MgO	85.592
SiO_2_	5.221
CaO	3.014
Fe_2_O_3_	1.824
Al2O_3_	1.239
SO_3_	0.251
Na_2_O	0.182
P_2_O_5_	0.138
MnO	0.113
Cr_2_O_3_	0.067
K_2_O	0.047
TiO_2_	0.037
Cl	0.017
SrO	0.007
CO_2_	2.25

**Table 2:** Mineral composition of the prepared sample (%), w/s = 10, the concentration of CsCl (C_CsCl_).

	MgO	KH_2_PO_4_	w/s	C_CsCl_
	(Not Raw MgO)			
MP1	0.5	0.5	10	0 M
MP3	0.75	0.25		
MP5	0.83	0.17		
MP7	0.875	0.125		
Cs1	0.5	0.5	10	0.02 M
Cs3	0.75	0.25		
Cs5	0.83	0.17		
Cs7	0.875	0.125		

**Table 3:** Initial distribution of ions and molecules in MD models.

Mg/P Ratio	Mg/P Ratio	0–4.21 Å	5–25 Å	25–45 Å
MP1	1	Solid matrix MgO containing 1200 Mg atoms and 1200 O atoms	2227 H_2_O	
			80 Mg^2+^	170 K^+^
			80 HPO_4_^2−^	80 HPO_4_^2−^
			\	10 H_2_PO_4_^−^
MP3	3	Solid matrix MgO containing 1200 Mg atoms and 1200 O atoms	3245 H_2_O	
			100 Mg^2+^	230 K^+^
			100 HPO_4_^2−^	100 HPO_4_^2−^
			\	30 H_2_PO_4_^−^
MP5	5	Solid matrix MgO containing 1200 Mg atoms and 1200 O atoms	4200 H_2_O	
			120 Mg^2+^	280 K^+^
			120 HPO_4_^2−^	120 HPO_4_^2−^
			\	34 H_2_PO_4_^−^
Cs1	1	Solid matrix MgO containing 1200 Mg atoms and 1200 O atoms	2227 H_2_O	
			80 Mg^2+^	2Cs^+^ and 168 K^+^
			80 HPO_4_^2−^	80 HPO_4_^2−^
			\	10 H_2_PO_4_^−^
Cs3	3	Solid matrix MgO containing 1200 Mg atoms and 1200 O atoms	3245 H_2_O	
			100 Mg^2+^	3Cs^+^ and 227 K^+^
			100 HPO_4_^2−^	100 HPO_4_^2−^
			\	30 H_2_PO_4_^−^
Cs5	5	Solid matrix MgO containing 1200 Mg atoms and 1200 O atoms	4200 H_2_O	
			120 Mg^2+^	4Cs^+^ and 276 K^+^
			120 HPO_4_^2−^	120 HPO_4_^2−^
			\	34 H_2_PO_4_^−^

**Table 4:** Immobilization rate of Cs^+^ in each group.

	Initial c_Cs+_ in Solution, Unit: M	Final c_Cs+_ in Solution, Unit: M	Ratio of c_Cs+_ in Solution	Ratio of c_Cs+_ in Solid
Cs1	0.02	18.5583 × 10^−5^	0.93%	99.07%
Cs3	0.02	3.22874 × 10^−5^	0.16%	99.84%
Cs5	0.02	2.54703 × 10^−5^	0.13%	99.87%
Cs7	0.02	3.42062 × 10^−5^	0.17%	99.83%

## Data Availability

Data are contained within the article.
